# Biological Evaluation of Oil-in-Water Microemulsions as Carriers of Benzothiophene Analogues for Dermal Applications

**DOI:** 10.3390/biomimetics6010010

**Published:** 2021-01-27

**Authors:** Ioanna Theochari, Tanja Ilic, Ines Nicolic, Vladimir Dobricic, Alia Tenchiou, Demetris Papahatjis, Snezana Savic, Aristotelis Xenakis, Vassiliki Papadimitriou, Vasiliki Pletsa

**Affiliations:** 1 Institute of Chemical Biology, National Hellenic Research Foundation, 48 Vassileos Constantinou Avenue, 11635 Athens, Greece; jtheohari@eie.gr (I.T.); tenchiu@eie.gr (A.T.); dpapah@eie.gr (D.P.); arisx@eie.gr (A.X.); vpapa@eie.gr (V.P.); 2 Department of Pharmaceutical Technology and Cosmetology, Faculty of Pharmacy, University of Belgrade, Vojvode Stepe 450, 11221 Belgrade, Serbia; tanja.ilic@pharmacy.bg.ac.rs (T.I.); ines.nikolic@pharmacy.bg.ac.rs (I.N.); snexs@pharmacy.bg.ac.rs (S.S.); 3 Department of Pharmaceutical Chemistry, Faculty of Pharmacy, University of Belgrade, Vojvode Stepe 450, 11221 Belgrade, Serbia; vladimir@pharmacy.bg.ac.rs

**Keywords:** drug delivery, cell viability, Franz diffusion cell, chemotherapy, skin permeation, differential tape stripping

## Abstract

During the last decade, many studies have been reported on the design and formulation of novel drug delivery systems proposed for dermal or transdermal administration. The efforts focus on the development of biocompatible nanodispersions that can be delivered to the skin and treat severe skin disorders, including cancer. In this context, oil-in-water (O/W) microemulsions have been developed to encapsulate and deliver lipophilic bioactive molecules for dermal application. An O/W biocompatible microemulsion composed of PBS buffer, Tween 80, and triacetin was assessed for its efficacy as a drug carrier of DPS-2, a lead compound, initially designed in-house to inhibit BRAF^V600E^ oncogenic kinase. The system was evaluated through both in vitro and ex vivo approaches. The cytotoxic effect, in the presence and absence of DPS-2, was examined through the thiazolyl blue tetrazolium bromide (MTT) cell proliferation assay using various cell lines. Further investigation through Western blotting revealed that cells died of necrosis. Porcine ear skin was used as a skin model to evaluate the degree of permeation of DPS-2 through skin and assess its retention. Through the ex vivo experiments, it was clarified that encapsulated DPS-2 was distributed within the full thickness of the stratum corneum (SC) and had a high affinity to hair follicles.

## 1. Introduction

During the last decade, a lot of efforts have focused on the design and development of novel drug delivery carriers to address drawbacks such as low solubility, stability and bioavailability in pharmaceutics and medicine [1,2,3]. A growing number of studies refer to the development of drug nanocarriers based on soft nanosized systems. These are nanoscale materials (10–200 nm) consisting of safe, biodegradable ingredients of relatively low cost that are capable of encapsulating the bioactive molecule at an effective dosage and designed in compliance with the desired biological and physicochemical properties [2,3]. Nanosized colloidal delivery systems, such as liposomes, polymers, nanoparticles, emulsions, and microemulsions, present many advantages in terms of the effective targeted delivery of hydrophobic bioactive compounds, mainly due to their high ratio of surface area to volume and their unique physicochemical properties [4,5,6]. The bioactive compounds are administered by such delivery systems to specific tissue or cell targets via oral, parenteral, topical, transdermal, nasal, and ocular routes [7,8]. Moreover, nanocarriers emerge as a promising approach for cancer chemotherapy. Despite the significant progress made in cancer treatment over the years, many metastatic sites are still not accessible due to the small size of malignant cell clusters and low chemosensitivity [9]. Most of the chemotherapeutic drugs that are currently available exhibit a poor water solubility, which leads to increased dosages and, consequently, toxic side effects. Therefore, efforts have focused on the development of nanoformulations and related delivery mechanisms in order to improve the bioavailability and efficacy of chemotherapeutic drugs [10].

Among nanosized colloidal delivery systems, pharmaceutical microemulsions are becoming very important as drug delivery systems due to their potential to incorporate a wide range of drug molecules, both hydrophilic and hydrophobic, and their promising applications in the field of cancer chemotherapy [10,11,12]. Microemulsions are low viscosity, optically transparent, thermodynamically stable dispersions of oil and water stabilized by surfactant molecules. They are isotropic in nature, with typical droplet diameters in the size range of 10–100 nm. They can be classified into three main categories according to the ratios of the dispersed phase in relation to the dispersion medium: oil-in-water (O/W), water-in-oil (W/O), and bicontinuous [13,14,15]. As they have gained researchers’ interest during the last decade, up to 1200 publications have reported biomedical drug delivery applications of microemulsions related to different routes of administration; oral delivery is suggested in over 42% publications, followed by dermal/topical delivery (>30%) and parenteral delivery (14.25%) [16].

**Figure 1:**
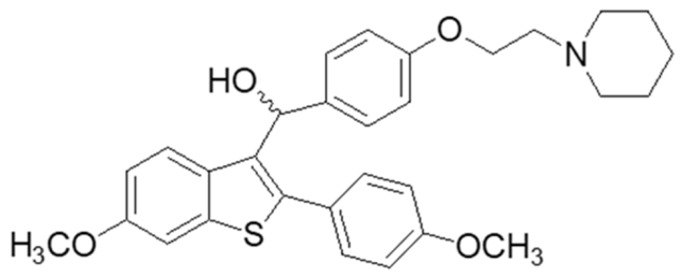
Chemical structure of DPS-2.

In this context, we developed and characterized O/W biocompatible microemulsions composed of PBS buffer, Tween 80, and triacetin [17] as carriers of the benzothiophene analogue DPS-2 (Figure 1) in skin melanoma (WM 164) and epithelial colorectal adenocarcinoma (Caco-2) human cell lines [18].

The encapsulated compound (DPS-2), initially designed to inhibit the BRAF^V600E^ oncogenic kinase, has not been proven to be a specific BRAF^V600E^ inhibitor but exhibited significant cytotoxic activity when encapsulated in O/W microemulsions and delivered in several colorectal cancer (CRC) and melanoma cell lines, affecting major proliferative/survival pathways [17,18,19]. The antitumor activity of DPS-2 has also been validated in vivo in mouse xenografts of Colo-205 colorectal cancer cells, where it remarkably reduced their tumor formation properties [19]. The above findings overall suggest that DPS-2 is a promising lead compound towards developing a novel chemotherapeutic agent.

Based on the above evidence, we further focused on dermal applications. The skin is the largest organ of the human body, accounting for about 15% of the total body weight, acting as a barrier between the internal and the external environment and providing protection against foreign bodies [20]. Many research groups report the enhancement of penetration and the increase in bioavailability of poorly soluble drugs when administrated through the skin [21,22,23,24,25,26,27,28]. Moreover, cancer treatment remains a major issue in dermal and transdermal applications. A variety of anticancer drugs and lead compounds, with potent anticancer activity, were examined for their efficacy when administrated by microemulsions [17,18,29,30]. Both microemulsions and microemulsion-based gels are frequently used in dermal and transdermal administration, providing an easy, convenient self-treatment for patients. The drug’s ability to penetrate through the skin is correlated to its physicochemical properties, as well as the pharmaceutical dosage form; furthermore, it is absolutely necessary to understand the processes, pathways, and driving forces affecting its transdermal permeation [31]. As it is not often possible to conduct in vivo studies to explore new routes of drug administration at the initial stages due to regulations or ethical concerns and it is nearly impossible to assess skin permeability using simply in vivo tests [31,32], ex vivo studies and relevant models are crucial in research on skin formulations for either topical or systemic drug actions [32]. Porcine ear skin is the most suitable ex vivo model for human skin in studies on dermal and/or transdermal penetration; thus, it is widely applied as a tool for topical formulation, evaluation, and optimization studies [33,34,35].

Therefore, in the present study, the developed O/W biocompatible microemulsion system was evaluated as a carrier of the cytotoxic lead compound DPS-2 in dermal applications through both in vitro and ex vivo approaches. The cytotoxic effect in the presence and absence of DPS-2 was examined through the thiazolyl blue tetrazolium bromide (MTT) cell proliferation assay using normal skin and melanoma cell lines. Further investigation of the mode of cell death, through Western blotting, confirmed that cells died of necrosis. Porcine ear skin was used as a skin model to evaluate the degree of permeation of DPS-2 through skin and assess its retention. The ex vivo approach, using the differential stripping technique, was focused on the identification of the main penetration route through which the encapsulated compound was delivered into the skin.

## 2. Materials and Methods

### 2.1. Materials

Triacetin (>98%) was purchased from TCI Chemical Industry Co., Ltd. (Tokyo, Japan). Polyoxyethylene sorbitan mono-oleate (Tween 80) suitable for cell cultures and dimethyl sulfoxide (DMSO) was purchased from Sigma-Aldrich (Taufkirchen, Germany). All cell lines used in this study were grown in EMEM medium supplemented with 10% FBS, antibiotics, and amino acids, all from Invitrogen. Eagle’s Minimum Essential Medium (EMEM), nonessential amino acids solution (100×), fetal bovine serum (FBS), L-glutamine (2 mM), bovine serum albumin (BSA), trypsin 0.25%, and phosphate-buffered saline (PBS) were purchased from Gibco-Life Technologies (Grand Island, NY, USA). Trypan Blue solution and thiazolyl blue tetrazolium bromide (MTT) were obtained from Sigma-Aldrich (Taufkirchen, Germany). DPS-2 was synthesized, as previously described, by the Group of Pharmaceutical Chemistry at the Institute of Chemical Biology (National Hellenic Research Foundation) [17].

PI/RNase staining solution was purchased from Cell Signaling Technology (Danvers, MA, USA); radio immunoprecipitation assay (RIPA) lysis buffer was purchased from Santa Cruz Biotechnology, Inc. (Heidelberg, Germany); and the Super Signal^TM^ West Oico PLUS chemiluminescent substrate (ECL) Western blotting detection reagent kit was purchased from Thermo Scientific (Rockford, IL, USA). Antibodies were purchased from Cell Signaling Technology (Danvers, MA, USA) and R&D Systems (Minneapolis, MN, USA). Absolute ethanol, 2-propanol, methanol, and acetonitrile (HPLC grade) were obtained from Thermo Fisher Scientific (Kandel, Germany). Porcine ears were kindly donated by a local provider in Serbia, as the ex vivo experiments were performed in the Faculty of Pharmacy, University of Belgrade, Belgrade, Serbia.

### 2.2. Cell Lines, Culture, and Treatments

The human skin normal fibroblasts WS1 and the human skin malignant melanoma cells SK-MEL 28 (BRAF^V600E^) were purchased from the American Type Culture Collection (ATCC, Manassas, VA, USA). Both cell lines were grown in EMEM (containing 2 mM L-glutamine, 1 mM sodium pyruvate, and 1500 mg/L sodium bicarbonate), supplemented with 10% FBS and 1% penicillin/streptomycin (Gibco-Life Technologies), at 37 °C in a humidified incubator with 5% CO_2_. The cells were maintained as a monolayer culture and trypsinized when they reached 70% confluence. Cells were treated with the bioactive compound either encapsulated in microemulsions or diluted in DMSO at the final concentration of 5.6 µM. The DMSO concentration in the medium did not exceed 0.2% *v*/*v*. Cells were harvested using 0.25% trypsin solution in PBS at 48 and 72 h after the treatment onset. Non-treated cells were used in all cases as a control.

### 2.3. Cell Survival and Cytotoxicity Assays

The inhibition of cell proliferation was assessed 48 and 72 h after treatment by MTT assay (M5655; Sigma-Aldrich) according to the manufacturer’s standard protocol. Cell viability was measured at 470 nm (690 nm for background) using a Safire II TECAN microplate reader (Grödig, Austria). Data were obtained by performing two independent experiments, and each was carried out in pentaplicate. Νon-treated cells were used in all cases as a control. Statistical analysis was performed by Student’s *t*-test.

Cytotoxicity was initially assessed 48 and 72 h after treatment by Trypan Blue (Sigma-Aldrich, St. Louis, MO, USA; T8154) according to the manufacturer’s standard protocol. The cell count and viability was determined using a Neubauer counter and trypan blue dye (50 μL + 50 μL cell suspension). Cytotoxicity of DPS-2 was determined from the equation: % live cells = unstained cells/(stained + unstained cells) × 100.

### 2.4. Western Blotting

Total protein extracts were prepared using RIPA lysis buffer. Cell lysates (25 µg protein) were resolved by sodium dodecyl sulfate polyacrylamide gel electrophoresis (SDS–PAGE) gel electrophoresis, electro-transferred onto nitrocellulose (0.2 µm), and blocked as previously described [17,36]. Overnight incubation of the nitrocellulose with primary antibody at 4 °C was followed by incubation with secondary anti-rabbit IgG antibody conjugated to horseradish peroxidase (HAF008; R&D Systems, Minneapolis, MN, USA) for 1.5 h at room temperature. Detection was achieved by the Super Signal^TM^ West Oico PLUS chemiluminescent substrate (Thermo Scientific, Rockford, IL, USA) kit. Primary antibodies against the following proteins were used at the indicated dilutions: PARP (46D11), rabbit mAb (Cell Signaling Technology-9532, Danvers, MA, USA), 1:1000; β-actin (cs-8457, β-actin (D6A8), rabbit mAb, Cell Signaling Technology), 1:1000; caspase-3 (D3RXY), rabbit mAb (cs-14220, Cell Signaling Technology), 1:1000; MLKL (D2I6N), rabbit mAb (cs-14993, Cell Signaling Technology), 1:1000; phospho-MLKL (ser358) (D6H3V), rabbit mAb (cs-14993, Cell Signaling Technology), 1:1000; GAPDH (14C10), rabbit mAb (cs-2118, Cell Signaling Technology), 1:1000.

### 2.5. Ex Vivo Permeation Study

Ex vivo permeation protocols were applied using porcine ear skin as a skin model to evaluate the DPS-2 in skin using modified Franz diffusion cells. In particular, each cell contains a donor compartment and a receptor compartment separated by the membrane. The permeation motif of DPS-2 from microemulsions was determined by calculating the percentage of the compound that permeated in the receptor compartment of the cell over time. The quantity of DPS-2 was determined using a liquid chromatography–mass spectrometry (LC MS-MS) procedure. Porcine ears, obtained immediately after slaughter (before scalding), were carefully washed under cold running water, blotted dry with a soft tissue, wrapped in the aluminum foil, and stored at −20 °C until use (within one month). To prepare skin membrane, after thawing at room temperature hair was shortened with an electric trimmer, to avoid destroying hair follicles. The full-thickness skin was removed from the cartilage using a scalpel and punched to the discs with a diameter of 25 mm. Concurrently, the trans-epidermal water loss (TEWL) (Tewameter^®^ TM 210; Couragep Khazaka, Koln, Germany) was measured before the experiment to check the skin barrier integrity. Skin was placed between the donor and receptor compartment and left to equilibrate for 30 min before the sample application. The temperature in the Franz cell waterbath remained constant at 32 ℃, as this is the mean temperature of the skin surface. The receptor chamber of the Franz cell device was filled with a mixture of 80% PBS 1× solution and 20% ethanol (chamber volume: 12 mL; effective diffusion area: 2.01 cm^2^) to guarantee that the DPS-2 permeation through the skin was not affected by the solubility parameters. The volume of the sample that was applied on skin surface was 500 μL.

According to the relevant guidelines, in order to test substances that penetrate slowly (OECD, 2004; EMA, 2018) a permeation study was performed for 30 h. Taking into account scheduling issues having to do with the laboratory infrastructure availability, the samples were collected from receptor chamber for analysis at 3, 6, 9, 27, and 30 h after treatment [37,38]. After the end of the experiment, the skin content was extracted to evaluate the quantity of DPS-2 that remained in full-thickness skin and did not permeate the skin barrier. In particular, skin samples were cleaned using PBS 1× solution and cut in small pieces to increase the extraction surface. Extraction was performed using methanol (HPLC grade) and samples were left to shake for 24 h. Then, samples were put in ultrasound bath for 15 min at room temperature and were centrifuged until the transparence of the supernatant was observed (3000 rpm, 15–30 min, 25 °C). Supernatant was stored at 4 °C until analysis and skin was discarded [39].

### 2.6. Differential Tape Stripping

Differential tape stripping using porcine ear skin contributed to the evaluation of the quantity of DPS-2 that penetrated through the superficial skin layers. [40]. Opposite to the in vitro permeation procedure where the skin was excised from the porcine ears, during this investigation the skin remained on the cartilage. Precisely, on the day of experiment, after defrosting, hairs were shortened and ears with intact skin were fixed on styrofoam plates. When the TEWL reached the value of approximately 15 g·m^−2^·h^−1^ (measured using a Tewameter^®^ TM 210) [40], the investigated formulation was carefully applied on assigned skin sites. The investigated microemulsion was applied in skin at an infinite dose regimen and left for 2 h (sample volume: 1 mL; effective diffusion area: 4.02 cm^2^). During this time, the mass of each adhesive tape was measured accurately before use. The TEWL was measured both before stripping as a baseline value and after the stripping of the 4th, 8th, and 12th tape to standardize the removed SC thickness. 

Adhesive tapes were pressed onto the skin using a roller and then removed. The procedure was repeated for 15 times. Afterwards, a drop of cyanoacrylate superglue was applied in the center of the treated site and was covered with an adhesive tape. The superglue was left for 10 min to polymerize and then the adhesive tape was removed, containing the follicular content of the drug. After the removal of the SC layers, each tape was placed into a centrifuge tube and drug was extracted by choosing a suitable solvent (70% ethanol for tapes containing SC and acetonitrile for tapes containing hair follicles). Then, samples were put in an ultrasound bath for 15 min at room temperature and centrifuged until the transparence of the supernatant was observed (4000 rpm, 15 min, 25 °C). Supernatant was kept at 4 °C and skin was discarded. The quantity of DPS-2 was determined using the LC MS-MS procedure.

### 2.7. Quantification of DPS-2

The liquid chromatography–mass spectrometry (LC MS-MS) procedure was used to quantify DPS-2 in all samples obtained through ex vivo permeation study and differential tape stripping. In the present study, samples that contained DPS-2 were analyzed on a Thermo Scientific Accela 1000 UPLC system coupled to a Thermo Scientific TSQ Quantum Access MAX triple quadrupole mass spectrometer. The analysis was performed on a UHPLC chromatograph ACELLA (Thermo Fisher Scientific Inc., Madison, WI, USA), coupled to a triple quadrupole mass spectrometer TSQ Quantum Access MAX (Thermo Fisher Scientific Inc., Madison, WI, USA) with a heated electrospray ionization (HESI) interface. The column was a Zorbax Eclipse XDB C18 (150 mm × 4.6 mm, 5 µm particle size). The mobile phase was acetonitrile/0.1% formic acid = 60:40 (*v*/*v*), the flow rate was 0.5 mL min^−1^, the column temperature was set to 30 °C, and the injection volume was 10 µL. DPS-2 was detected and quantified in positive HESI mode (*m*/*z* = 504.4).

## 3. Results

### 3.1. In Vitro Evaluation

#### 3.1.1. Cell Proliferation and Cytotoxicity Assays

**Figure 2:**
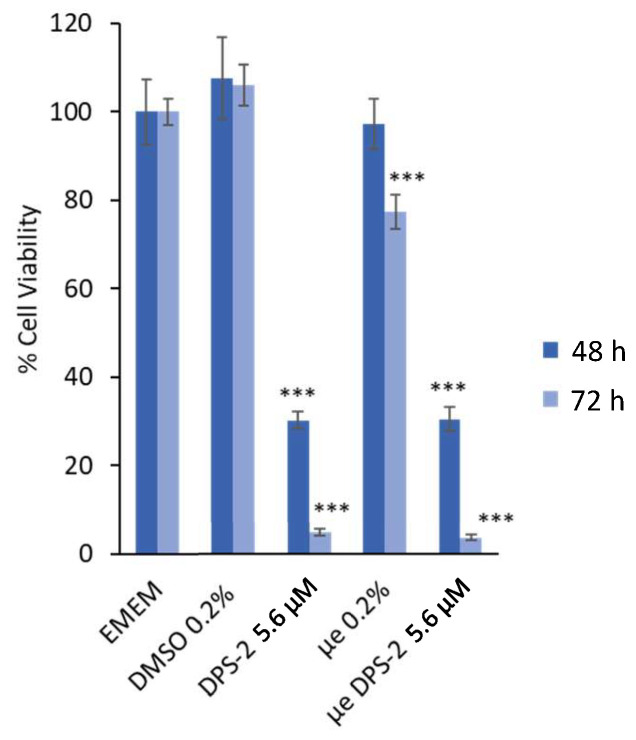
Inhibition of cell proliferation in SK-MEL 28 cells 48 and 72 h after the treatment onset. Culture medium (EMEM) and DMSO 0.2% *v*/*v* were used as positive control samples, μe 0.2% refers to O/W microemulsions without DPS-2 (empty), μe DPS-2 5.6 μΜ refers to O/W microemulsions loaded with DPS-2 at a concentration of 5.6 μM. DPS-2 was also solubilized in DMSO at 5.6 μΜ. Statistically significant results are indicated by asterisks (***) when *p* < 0.001 (Student’s *t*-test).

**Figure 3:**
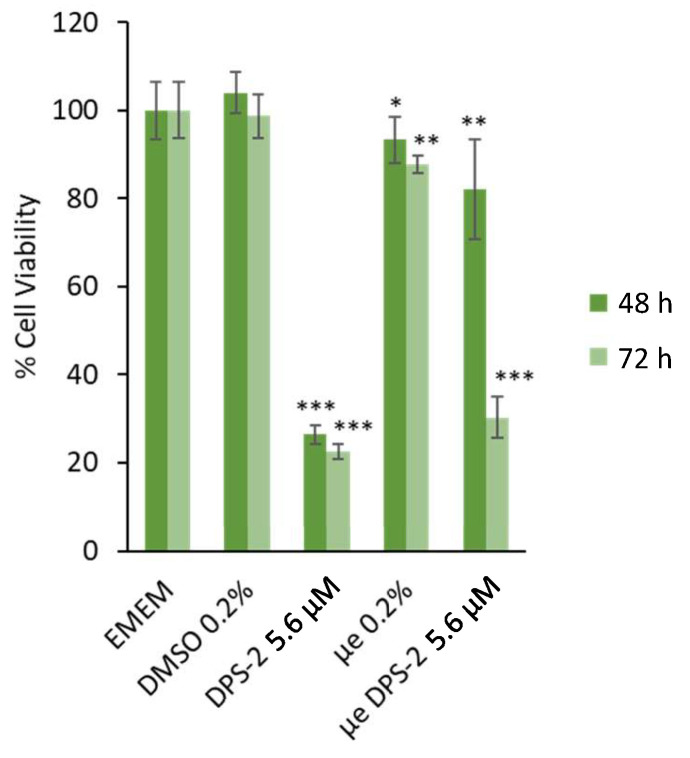
Inhibition of cell proliferation in WS1 cells 48 and 72 h after the treatment onset. Culture medium (EMEM) and DMSO 0.2% *v*/*v* were used as positive control samples, μe 0.2% refers to O/W microemulsions without DPS-2 (empty), μe DPS-2 5.6 μΜ refers to O/W microemulsions loaded with DPS-2 at a concentration of 5.6 μM. DPS-2 was also solubilized in DMSO at 5.6 μΜ. Statistically significant results are indicated by asterisks (*) when *p* < 0.05, (**) when *p* < 0.01, and (***) when *p* < 0.001 (Student’s *t*-test).

Cell viability data are very important as the in vitro assessment of different surfactants or surfactant mixtures in drug delivery systems is required for the investigation of potential skin irritation and cytotoxic effects [41,42]. The inhibition of cell proliferation was determined using the MTT assay, a method for the sensitive quantification of viable cells. As shown in Figure 2 and Figure 3, the lipophilic bioactive compound DPS-2 was either diluted in DMSO or encapsulated in O/W microemulsions and was administered in human skin malignant melanoma cells (SK-MEL 28) and normal fibroblasts (WS1).

Prior to treatment, empty and loaded microemulsions were diluted in the cell culture medium (EMEM) at a ratio of 0.2% *v*/*v*, as previously described [17,19]. The final concentration of DPS-2 in cell culture medium was 5.6 μΜ, either encapsulated in microemulsion or diluted in DMSO. Figure 2 and Figure 3 show the percentage of cell viability in SK-MEL 28 and WS1 cell cultures, respectively, 48 and 72 h after the treatment onset. EMEM and DMSO 0.2% *v*/*v* were used as positive control samples in all cases.

The in vitro evaluation data revealed that the O/W microemulsions loaded with DPS-2 significantly inhibited cell proliferation at 48 and mainly 72 h in both cell lines. In accordance with previously made observations [18], empty O/W microemulsions at a final ratio of 0.2% *v*/*v* in the culture medium slightly affected cell viability, most likely due to the transient disturbance of the cellular membrane lipid bilayer in the course of their penetration within cells [17,43]. Nevertheless, it is important to note that the cytotoxic effect of O/W microemulsions loaded with DPS-2 was prominent at both time points and far more prominent in SK-MEL 28 melanoma cells compared to WS1 normal skin fibroblasts. In any case, the inhibition of cell proliferation induced by O/W microemulsions loaded with DPS-2 was comparable to the one observed when DPS-2 was administered via DMSO, with the exception of 48 h in normal skin fibroblasts (WS1), which is probably due to the slower proliferation rate characterizing normal cells and, hence, the delayed manifestation of the cytotoxic effect observed at 72 h. Overall, the effect of the loaded O/W microemulsions, in terms of the inhibition of cell proliferation and cytotoxicity, was evidently differentiated between melanoma and normal cells.

Subsequently, the % cell death was assessed by trypan blue exclusion assay in WS1 (Table 1) and SK-MEL 28 (Table 2) cells grown in 6-well plates and trypsinized 72 h after the treatment onset, as previously described [17]. In accordance with the MTT results, only in SK-MEL 28 cells treated with DPS-2 was the % cell death was considerably increased (Table 2).

#### 3.1.2. Molecular Analysis by Western Blotting

**Figure 4:**
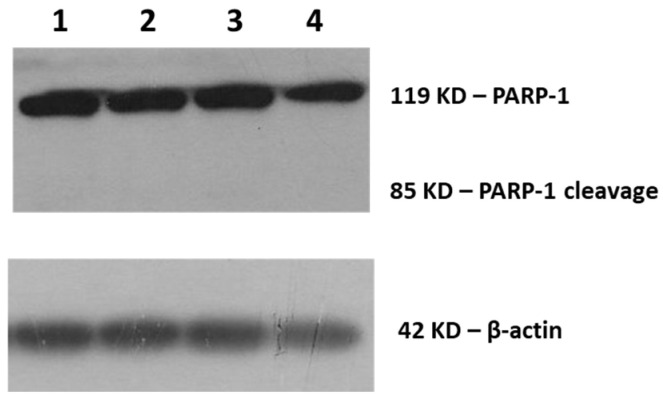
Cleavage of poly(adenosine diphosphate-ribose) polymerase 1 (PARP-1) for the assessment of cell death. Immunoblot of SK-MEL 28 cell extracts (25 μg) with anti-PARP-1 antibody detecting the 85 kDa PARP fragment (cleav. PARP-1) and the 119 kDa intact PARP-1 form. Cells were either not treated (EMEM-1) (**1**), treated with DPS-2 solubilized in DMSO at 5.6 μΜ (**2**), or treated with O/W microemulsion empty 0.2% *v*/*v* (**3**) or O/W microemulsion loaded with DPS-2 at 5.6 μM (**4**) and maintained in culture for 72 h. The relative abundance of the total protein applied was measured by using the amount of actin as a control, as assessed by the anti-β-actin on the same blot.

The biochemical analysis of cell death markers was performed by Western blotting as previously described [18]. The specific cleavage of poly(adenosine diphosphate-ribose) polymerase 1 (PARP-1), a nuclear protein implicated in DNA repair, to a 89 kDa fragment as a result of the caspase protease activity associated with the activation of apoptosis is considered as a sensitive apoptotic marker [44]. As shown in Figure 4, PARP-1 cleavage was not detected in SK-MEL 28 cells treated with DPS-2.

**Figure 5:**
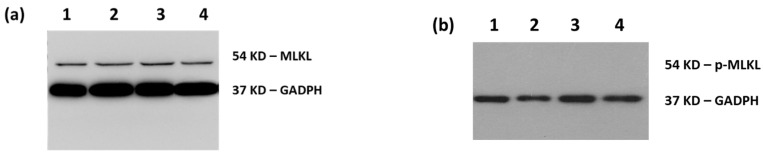
Immunodetection of necroptotic cell death markers. Phosphorylation of mixed lineage kinase domain-like protein (MLKL) at ser358. Immunoblot of SK-MEL 28 cell extracts (25 μg) with (**a**) anti-MLKL antibody detecting all forms of the MLKL protein (54 kDa) and (**b**) anti-phosho-MLKL antibody detecting the phosphorylation of MLKL at serine 358 (54 kDa) upon the activation of necroptosis. Cells were either not treated (EMEM-1) (1), treated with DPS-2 solubilized in DMSO at 5.6 μΜ (2), or treated with O/W microemulsion empty 0.2% *v*/*v* (3) or O/W microemulsion loaded with DPS-2 at 5.6 μM (4) and maintained in culture for 72 h. The relative abundance of total protein applied was measured by using the amount of GAPDH as a control, as assessed by the anti-GAPDH on the same blot.

The additional immunoblotting of the apoptotic marker caspase-3 [45] (data not shown), as well as necroptotic markers mixed lineage kinase domain-like protein (MLKL) and phospho-MLKL (Figure 5), confirmed that SK-MEL 28 cells did not die through a programmed cell death mode but eventually of necrosis [46,47].

### 3.2. Ex Vivo Evaluation

#### 3.2.1. Ex Vivo Permeation Study

An ex vivo permeation protocol was applied to determine the quantity of DPS-2 passing into the receiving compartment as well to assess its retention in the skin. In other words, the experimental settings used enabled the evaluation of DPS-2 skin uptake, as well as the assessment of the microemulsion’s potential to target skin. Permeability experiments were carried out using modified Franz diffusion cells, and porcine ear skin was used as the selected biological membrane. The permeated quantity of DPS-2 was determined using LC-MS/MS and the samples were taken at 3, 6, 9, 27, and 30 h after administration. The study was performed in three independent experiments, each time in duplicate, and the total amount of porcine ear skin samples tested was six.

DPS-2 was not detected in samples taken from receptor’s compartment in any time point during the study—i.e., the amount of DPS-2 in the receptor medium was below the LC MS-MS detection limit. On the other hand, DPS-2 was detected in all skin extracts obtained at the end of the experiment—i.e., 30 h after the administration. Precisely, the total quantity of DPS-2 that was deposited in the full-thickness porcine ear skin within 30 h was determined as 10.92 ± 7.55 μg/cm^2^. The obtained relatively high standard deviation was highly expected and is frequently reported in the literature, since porcine ear skin was used as the biological membrane. It is well known that it is difficult to control the breeding and diet conditions of pigs, which can affect, inter alia, the skin properties (the ears are usually obtained from local abattoir immediately after slaughter). Here, it is important to note that the ex vivo permeation test was performed under an infinite-dosing regimen (the alterations in the donor chamber caused by evaporation and diffusion were negligible) to achieve steady-state conditions. The ex vivo permeation results obtained unequivocally indicate that DPS-2 is predominately located within the full-thickness skin, implying that the proposed O/W microemulsion is suitable to be used for the skin targeting of lipophilic bioactive compounds, such as DPS-2.

#### 3.2.2. Differential Tape Stripping

**Figure 6:**
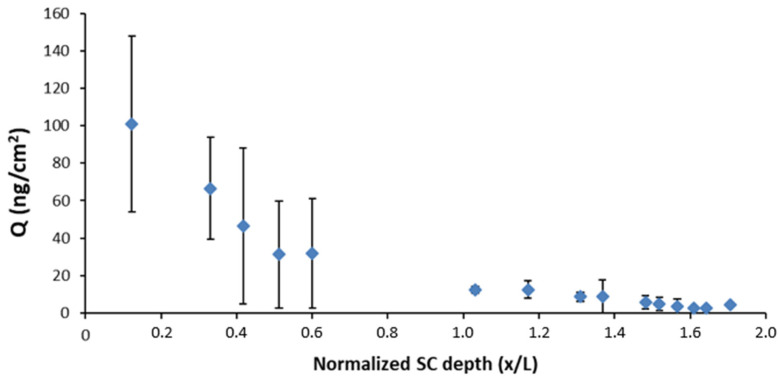
Representation of the quantity of DPS-2 (ng/cm^2^) in relation to the normalized SC depth.

Differential Tape Stripping was performed on porcine ear skin to quantify the amount of DPS-2 that had penetrated the SC layer and hair follicles. Thus, this method enabled the identification of the preferable penetration pathway(s) contributing to DPS-2 delivery to the skin via the developed O/W microemulsion. Although there are specific differences in anatomy among human and porcine hair follicles, several similarities are also identified. Thus, the ex vivo model of porcine ear is an applicable and reliable model to evaluate the penetration of bioactive compounds in SC and hair follicles [48]. The quantity of DPS-2 was detected using LC-MS/MS and samples were taken 2 h after the administration of the loaded O/W microemulsion under infinite dose conditions. The study was performed in five independent experiments. Here, it is important to note that we firstly performed a preliminary study to determine the number of tape strips required in order to completely remove the DPS-2 localized in the SC. The results obtained revealed that fifteen (15) tapes were enough to remove the DPS-2 from the SC. As observed in Figure 6, the last tape contained almost 2 ng/mL, which was the lowest detection limit (LOD) of LC MS/MS used in this study. Hence, the quantity of DPS-2 extracted from the cyanoacrylate biopsies can be defined as the quantity present in hair follicles.

DPS-2 was detected in all samples taken from SC in decreasing concentrations as the SC layers were removed (Figure 6) with an increasing tape number. The relatively high variability of data obtained for the first five tapes could be ascribed to the specific structure of porcine ear skin, the inherent variability between porcine ear skin thicknesses, and the influence of the tested sample affecting the amount of SC (corneocyte clusters) removed through the tape stripping procedure. Precisely, the irregular surface structure of the porcine skin with loose cell clusters and canyons has been described in the literature and could be responsible for the variable amount of SC removed with the first adhesive tapes. Additionally, the presence of residual humidity and microemulsion components in the surface layers of SC can affect tape tackiness—i.e., the ability of the tapes to adhere to the skin surface properly. On the other hand, the lower variability of data obtained with an increasing tape number could be attributed to the increasing corneocyte adhesion in the deeper SC layers and an increasingly homogeneous structure [49]. The results of the cyanoacrylate biopsies revealed that DPS-2 penetrated into the hair follicles to a considerable extent (a quantity of 72.9 ± 45.5 ng/cm^2^ reached the hair follicles, while a quantity of 292.4 ± 101.6 ng/cm^2^ was detected in the SC). This finding clearly implies that the transfollicular pathway has an important role in the overall skin penetration of DPS-2 carried by the tested O/W microemulsions.

## 4. Discussion

During the last five years, researchers have reported promising results in developing pharmaceutically accepted O/W microemulsions as delivery systems of lipophilic drugs for oral or topical administration [1,2,50]. Such biocompatible microemulsion formulations could be extremely effective in the selective delivery of chemotherapeutics to cancer cells, a major issue in cancer research worldwide [2,51].

Therefore, pharmaceutically applicable microemulsions based on Tween 80, triacetin, and PBS buffer solution were previously developed as matrices for the encapsulation of DPS-2, a novel lipophilic benzothiophene analogue. Due to its potent cytotoxic activity in various human cancer cell lines, DPS-2 is currently being developed as a new lead compound in drug design [17,18]. In a previous study, the developed O/W microemulsions were subjected to two different analytical techniques, Dynamic Light Scattering (DLS) and Electron Paramagnetic Resonance (EPR) spectroscopy, to assess their structural characteristics and also the influence of the drug on the microstructure [17,19]. Following the structural characterization, the developed microemulsions, empty as well as loaded with DPS-2, were submitted to in vitro biological evaluation in MW 164 skin melanoma and Caco-2 human epithelial colorectal adenocarcinoma cell lines. The choice of these cancer cell lines was justified by the use of microemulsions for transdermal and oral drug delivery [2,52,53,54]. The proposed O/W microemulsions were proven suitable as carriers of DPS-2 into human cancer cell lines and DPS-2 exerted its cytotoxic activity through stalling and, finally, collapse of the replication fork [17]. It is worth mentioning that DPS-2 encapsulated in microemulsions showed a response comparable to the one observed when the compound, at the same concentration (5.6 μM), was administered via DMSO, commonly used as solvent and carrier of small molecules through cell membranes [17,19].

Guided by the encouraging results on WM 164 melanoma cell lines, in the context of the present work, we focused on dermal/transdermal applications as effective skin cancer treatment remains a major challenge. A variety of anticancer drugs and lead compounds, with potent anticancer activity, have already been examined for their efficacy when administrated by microemulsions [17,19,29,30]. Therefore, following in vitro evaluation in the human skin normal fibroblasts WS1 and the malignant melanoma cells SK-MEL 28, the previously developed O/W biocompatible microemulsion system was evaluated as DPS-2 carrier in potential dermal/transdermal delivery through the porcine ear skin ex vivo model.

As shown in Figure 2 and Figure 3, DPS-2 encapsulated in O/W microemulsions at 5.6 μM affected the cell proliferation rate in both cell lines tested at 72 h, however, the inhibition of cell proliferation in SK-MEL 28 melanoma cells was far more prominent compared to WS1 skin normal fibroblasts. This is in line with previous results observed in WM 164 melanoma cells and the compound’s mechanism of cytotoxic action through stalling and collapse of the replication fork, according to which, it is expected to kill rapidly proliferating cancer cells more effectively. In order to confirm that cells died of necrosis, as previously shown [17], the immunoblotting of several markers of programmed cell death [42,43,44,45] was subsequently performed. Indeed, as shown in Figure 4 and Figure 5, the cell death induced by DPS-2 encapsulated in O/W microemulsions in SK-MEL 28 cells was neither apoptotic nor necroptotic. DPS-2 has been proven a potent cytotoxic agent in a wide range of cancer cell lines so far [17,18], among them melanoma cell lines which are often extremely resistant to cytotoxic agents due to a heavy burden of critical mutations in cell proliferation/death pathways and severe genomic instability [55]. Drug’s encapsulation in the oil cores of microemulsions, being effective in terms of cytotoxicity, could pave the way for its use in targeted, topical delivery against skin cancers, in combination with surgical excision or whenever surgery is not an option [56].

The ex vivo model of porcine ear is a suitable and reliable model to evaluate the penetration of bioactive compounds into the skin. As described above, the permeability motif of DPS-2 was clarified by determining its quantity in cell receptor compartment during time, using Franz diffusion cell. Under the experimental setting used, DPS-2 was not detected in all samples taken from receptor’s medium and was predominantly located within the full-thickness skin. This finding indicates the low risk for systemic absorption of DPS-2 and consequently, the compound is not likely to be distributed to other tissues, causing side effects. In other words, if DPS-2 develops into a potent chemotherapeutic agent against skin cancer, the proposed O/W microemulsion is suitable to be used for targeted skin delivery. Here, it is interesting to note that in the literature there is certain evidence that SC and epidermis are more lipophilic compared to other skin layers such as the dermis [57]. Based on this statement, the higher affinity of a lipophilic compound, such as DPS-2 (logP = 6.489) could be expected for the SC and epidermis compared to the dermis. However, this assumption requires further experimental confirmation.

Differential tape stripping revealed information on both the amount of DPS-2 that penetrated through the surface layers of the skin and the affinity of DPS-2 to the hair follicles. DPS-2 was detected in all samples taken from SC in decreasing concentrations. Likewise, a relatively high amount of DPS-2 was found in the hair follicles. Namely, the amount of DPS-2 in the hair follicles was only four times lower compared to the amount deposited in the SC. Recently, our research group has investigated the penetration of aceclofenac into the hair follicles from nanoemulsions using the same methodology. We observed that the amount of aceclofenac in hair follicles was more than 10 times lower compared to the amount deposited in the SC. The findings of the current work clearly confirm that the transfollicular pathway has an important role in the overall skin penetration of DPS-2 carried by the tested microemulsion. There are many reports suggesting that microemulsions can be delivered more efficiently and more deeply into the hair follicles compared to conventional formulations (solutions, creams). This might be due to the very low interfacial tension of microemulsion leading to an outstanding contact with the skin surface, in that way the microemulsion might be able to reach the interior of the follicles more easily [58,59,60]. Recently, Subongkot and Sirirak (2020) have observed that drug-loaded microemulsion particles penetrate into the hair follicles before releasing the entrapped drug [59]. In this context, it is important to emphasize that no transfollicular penetration of nanoparticles through the barrier of the hair follicles into the living epidermis has been demonstrated so far [60]. However, owing to specific structure of hair follicles (SC is only present in the upper part), the drug released from the microemulsion can independently translocate into the viable tissues, epidermis, and dermis more efficiently.

Several factors are involved in the penetration of the skin by DPS-2 carried within the oil core of the developed O/W microemulsion: (1) the low surface tension of the microemulsion, and, consequently, the close and prolonged contact with the skin, ensuring high concentration gradient for drug skin uptake; (2) the improved penetration of the microemulsion into the hair follicles, leading to improved delivery into viable skin layers (epidermis and/or dermis); (3) the perturbation of SC lipid matrix by interactions of the microemulsion’s components with the SC, leading to improved permeability through the SC lipid bilayers [59]. Actually, numerous studies have recently confirmed that, owing to relatively high surfactant concentration, microemulsions lead to alterations in skin barrier function, such as the disruption of intercellular regions of SC, due to the fluidization and/or extraction of the SC lipid matrix [61,62,63]. Dermal irritation is one of the most common adverse effects when applying formulations topically, therefore, when developing novel topical formulations, apart from efficacy, the assessment of the dermal irritation potential is a critical factor. In view of the development of DPS-2 to an anticancer formulation for topical administration when encapsulated in O/W microemulsions, further thorough investigation of the degree of disturbance of skin barrier integrity by the O/W microemulsion components, particularly during prolonged use, will be undertaken in our next study.

## 5. Conclusions

O/W nanodispersions based on safe materials were formulated as effective delivery vehicles of bioactive compounds of pharmacological interest. DPS-2, a potent cytotoxic benzothiophene analogue in a wide range of cancer cell lines, was encapsulated, as it is currently being developed as a new lead compound in drug design. Following the in vitro validation of the system as to its potent cytotoxicity in melanoma cell lines compared to normal skin fibroblasts, the ex vivo porcine ear skin approach was applied to evaluate the penetration of the encapsulated DPS-2 in SC. Through the ex vivo model, it was clarified that encapsulated DPS-2 was distributed within full-thickness skin and performed with a relatively high affinity to hair follicles.

In conclusion, the developed O/W nanodispersions could contribute to overcoming major problems related to administration through the skin barrier and the low water solubility of lipophilic compounds, enhancing their penetration through skin layers. Data revealed that DPS-2 is retained in the skin, which indicates that the developed nanoformulation is not suitable for transdermal delivery. However, the evidence generated in this study paves the way for the development of nanoformulations based on biocompatible colloids being used for the targeted delivery of lipophilic anticancer drugs against skin cancer, including melanoma.

## Figures and Tables

**Table 1:** Results of the cell death Trypan Blue assay using the WS1 cell line 72 h after the treatment onset.

Sample	% of Cell Death	±SD
Not treated	4.0	0.4
DMSO/DPS-2 5.6 μM	6.9	0.9
O/W microemulsion empty	3.6	0.6
O/W microemulsion loaded/DPS-2 5.6 μM	12.2	0.6

**Table 2:** Results of the cell death Trypan Blue assay using the SK-MEL 28 cell line 72 h after the treatment onset.

Sample	% of Cell Death	±SD
Not treated	8.7	0.5
DMSO/DPS-2 5.6 μM	9.8	0.4
O/W microemulsion empty	9.0	0.6
O/W microemulsion loaded/DPS-2 5.6 μM	18.7	1.1

## Data Availability

Not applicable.
